# Brilliant blue G, a P2X7 receptor antagonist, attenuates early phase of renal inflammation, interstitial fibrosis and is associated with renal cell proliferation in ureteral obstruction in rats

**DOI:** 10.1186/s12882-020-01861-2

**Published:** 2020-05-29

**Authors:** José Monteiro Sad Pereira, André Luis Barreira, Conrado Rodrigues Gomes, Felipe Mateus Ornellas, Débora Santos Ornellas, Luiz Carlos Miranda, Lucio Ronaldo Cardoso, Robson Coutinho-Silva, Alberto Schanaider, Marcelo M. Morales, Maurilo Leite, Christina Maeda Takiya

**Affiliations:** 1 Programa de Pós-Graduação em Ciências Cirúrgicas, Departamento de Cirurgia, Faculdade de Medicina, Universidade Federal do Rio de Janeiro, Rio de Janeiro, Brazil; 2 Serviço de Urologia, Hospital Universitário Clementino Fraga Filho, Universidade Federal do Rio de Janeiro, Rio de Janeiro, Brazil; 3 Serviço de Nefrologia, Hospital Universitário Clementino Fraga Filho, Universidade Federal do Rio de Janeiro, Rio de Janeiro, Brazil; 4 Instituto de Biofísica Carlos Chagas Filho, Universidade Federal do Rio de Janeiro, Rio de Janeiro, Brazil; 5 Centro de Cirurgia Experimental, Departamento de Cirurgia, Faculdade de Medicina, Universidade Federal do Rio de Janeiro, Rio de Janeiro, Brazil

**Keywords:** Renal inflammation, P2X7 receptor, Unilateral ureteral obstruction, Macrophages

## Abstract

**Background:**

Previous study showed that purinergic P2X7 receptors (P2X7R) reach the highest expression in the first week after unilateral ureteral obstruction (UUO) in mice, and are involved in the process of inflammation, apoptosis and fibrosis of renal tissue. We, herein, document the role of purinergic P2X7 receptors activation on the third day of UUO, as assessed by means of BBG as its selective inhibitor.

**Methods:**

We investigated the effects of brilliant blue G (BBG), a P2X7R antagonist, in the third day of kidney tissue response to UUO in rats. For this purpose, male Wistar rats submitted to UUO or sham operated, received BBG or vehicle (V), comprising four groups: UUO-BBG, UUO-V, sham-BBG and sham-V. The kidneys were harvested on day 3 UUO and prepared for histology, immunohistochemistry (P2X7R, PCNA, CD-68, α-sma, TGF-β1, Heat-shock protein-47, TUNEL assay), quantitative real-time PCR (IL-1β, procollagens type I, III, and IV) for mRNA quantification.

**Results:**

The group UUO-V presented an enhancement in tubular cell P2X7-R expression, increase influx of macrophages and myofibroblasts, HSP-47 and TGF- β1 expression. Also, upregulation of procollagen types I, III, and IV, and IL-1β mRNAs were seen. On the other hand, group UUO-BBG showed lower expression of procollagens and IL-1β mRNAs, as well as less immunoreactivity of HSP-47, TGF-β, macrophages, myofibroblasts, and tubular apoptosis. This group also presented increased epithelial cell proliferation.

**Conclusion:**

BBG, a known highly selective inhibitor of P2X7R, attenuated renal inflammation, collagen synthesis, renal cell apoptosis, and enhanced renal cell proliferation in the early phase of rat model of UUO.

## Background

Adenosine triphosphate (ATP) is a multifunctional nucleotide, released by injured/dying cells, and is the principal agonist for purinergic P2 receptors [1]. These receptors are divided into metabotropic G protein-coupled P2Y (P2YR) and ionotropic ligand gated P2X (P2XR). P2X are ligand-gated ion channels for Na+, Ca + and K+, known as ionotropic. Currently, seven subtypes of P2X receptors have been cloned and identified as P2X1–7 [2]. P2X7R are ATP-gated nonselective ion channels, permeable to Na+, K+, and Ca2+, expressed in a wide range of epithelial, endothelial, mesenchymal and immune cells. They are ubiquitously expressed in cortex and medulla, in vascular and tubular compartments [3]. P2X7R is scantly expressed in renal tissue in normal conditions, but can be upregulated in disease states [4, 5]. P2XR-ATP axis is important in homeostasis of diverse physiological and pathophysiological processes, including hypertension [6], diabetes [7, 8], polycystic kidney disease [9], inflammatory, and autoimmune disorders [10]. The activation of P2X7Rs may be involved in renal diseases and are widespread in renal compartments, expressed in immune cells, fibroblasts and myofibroblasts, upregulated in inflammation, and associated with the production of pro-inflammatory mediators [11, 12].

The progression of chronic kidney disease (CKD) is related to the intensity of renal interstitial fibrosis, the accumulation of extracellular matrix proteins and the process of renal cell death [13]. The importance of P2X7R in renal tissue fibrosis has been highlighted on P2X7R knockout mice submitted to UUO, a well-known model of tubulointerstitial fibrosis [5].

In the present study, we attempted to investigate the effect of BBG, a selective P2X7R antagonist, on the early development of renal injury after UUO in rats, in order to better elucidate the role of purinergic signaling antagonism on the processes of renal inflammation, fibrosis, renal cells apoptosis and regenerative proliferation in this setting.

## Methods

Forty male adult Wistar rats were housed under specific pathogen-free conditions, with controlled temperature and relative humidity, and provided standard rat chow and water ad libitum. This study was approved by the Animal’s Ethics Committee from the Health Sciences Center, Federal University of Rio de Janeiro and is in compliance with the guidelines as recommended by the National Research Council’s criteria (NIH No. 86–23).

### Experimental protocol

Rats were randomly divided into four groups of 5 animals each. Two groups were submitted to a complete UUO and received BBG (UUO-BBG) or vehicle (UUO-V). The other two groups, SHAM-operated, received BBG or vehicle (SHAM-BBG or SHAM-V, respectively).

### Surgical procedure

Animals were anesthetized with ketamine (35 mg/kg) and xylazine (9 mg/kg) by peritoneal route. An abdominal midline incision was done and the left ureter was ligated at two points using 4–0 silk and sectioned. BBG (Brilliant Blue G), 40 mg/kg (Sigma-Aldrich, Saint Louis, MO, USA, cat. B0770) was dissolved in 0.2% dimethyl sulfoxide (DMSO, Sigma-Aldrich, cat. D2650) in sterile saline solution and was injected in the inferior cava vein (0.5 mL) after ureteral ligation (Group UUO-BBG). In another group, obstructed rats received vehicle instead of BBG (Group UUO-V). The abdominal wall was closed layers and the rats were kept in regular cages. The sham-operated animals underwent identical surgical procedures, but without ligation and sectioning of the left ureter.

### Tissue preparation

After 3 days following the surgical procedure, animals were sacrificed under anesthesia by means of cardiac puncture and perfusion in a controlled flow rate of 10 mL/min of a sterile 0.9% saline solution with heparin (5 U/mL), infused through the left ventricle to clean the blood vessels. The left kidney was stored for further analysis.

### Histopathological and Immunohistochemical studies

The kidneys were embedded in paraffin after fixation, sectioned in 3-μm width slices and stained for picro-sirius red (PS) assuming collagen quantification. Immunohistochemical procedures were performed on paraffin-embedded kidney sections. After dewaxing and rehydrating, sections were submitted to endogenous peroxidase inhibition. Heat mediated-antigen retrieval and enzymatic techniques were performed according to the specific antibody. After blocking the nonspecific binding of immunoglobulins to the tissue, primary antibodies were incubated overnight at 4 °C in a humidified chamber for about 16 h. The secondary antibodies were incubated (Histofine® Simple Stain Rat MAX - PO (Mouse), and - PO (Rabbit) from Nichirei, Japan). The chromogen substrate was diaminobenzidine (Liquid DAB, Dako, cat. K3468). For P2X7R staining, P2X7R antibody incubation was performed using the P2X7R peptide (control antigen for APR-004, Alomone) for 1 h at room temperature. This antibody solution was incubated on sections instead of the P2X7R antibody alone.

### Histomorphometry

Images were captured by a light microscope (Eclipse E800, Nikon, Japan) coupled to a digital camera and analyzed using the Image Pro Plus software (version 4.5.1, Media Cybernetics, Rockville, MD, USA).

### Picro-sirius red, P2X7R, macrophage (CD68), myofibroblast (α-SMA), heat shock protein 47 (HSP-47), and TGF-β1 surface density quantifications

Tissue sections were used to obtain 20 randomly chosen photomicrographs from renal cortex and medulla, avoiding fields containing blood vessels and glomeruli. Objective lens with a magnification of 40x was used. Results were expressed as surface densities in the cortex or medulla and were expressed as percentages mean ± standard error of the mean (SEM).

### Collagen content, and IL-1β in renal tissue by quantitative real-time reverse transcription-polymerase chain reaction (qRT-PCR)

Total ribonucleic acid (RNA) was extracted from snap frozen renal tissues using the spin total RNA Isolation System (Promega Corporation, Fitchburg, WI, USA) following manufacturer’s recommendations. RNA concentration was measured by spectrophotometry in Nanodrop ND-1000. First-strand cDNA was synthesized from total RNA using GoTaq 2-STEP RT qPCR System (Promega Corporation). The primers sequences for collagen content, and IL-1β are depicted in Table 1. Relative messenger RNA (mRNA) levels were measured with a SYBR green detection system using Mastercycler RealPlex 2 (Eppendorf, Hamburg, Germany). Samples were measured in triplicate. Ciclophilin was used as internal control.

**Table 1:** Base sequences of primers for Procollagens I, II and IV, IL-1β and ciclophilin

Genes	Sense	Anti-sense
Procollagen I	5’TGGAATCTTGGATGGTTTGGA 3’	5’ GCTGTAAACGTGGAAGCAAGG 3’
Procollagen III	5’ ACCTGGACCACAAGGACAC 3’	5’ TGGACCCATTTCACCTTTC 3’
Procollagen IV	5’ ATTCCTTTGTGATGCACACCAG 3’	5’ AAGCTGTAAGCATTCGCGTAGA 3’
IL-1β	5′-CTATGTCTTGCCCGTGGAG-3	5′-CATCATCCCACGAGTCACA-3′
Ciclophilin	5′-TCCACTTCGATCTTGCCACAGTCT-3’	5′-AGACACCAATGGCTCCCAGTTCTT-3′

### Renal cells apoptosis

Apoptotic tubular cells in kidney tissue were detected by the terminal deoxytransferase uridine triphosphate nick end-labeling technique (TUNEL), using ApopTag® Peroxidase in situ detection kit (Chemicon International, Temecula, CA, USA). The reaction was performed according to the manufacturer’s instructions, revealed with diaminobenzidine (Liquid DAB, Dako). Results were expressed as the percentage of positive tubular cells in a total of 100 cells.

### Proliferation index

Proliferation index was obtained by the ratio of tubular cells positive for PCNA (Proliferating cell nuclear antigen), divided by the total number of tubular cells. Results were expressed as the percentage of positive tubular cells.

### Statistical analysis

The results obtained from 20 randomly chosen photomicrographs of each animal, in each immunohistochemical study, presented as normal distribution. Results are expressed as mean ± SEM. Statistical analysis was performed using One-way ANOVA followed by the Tukey test. *P* < 0.05 was considered statistically significant.

## Results

### Histopathological study

Hematoxylin-eosin-stained sections of UUO animals showed interstitial enlargement and dilated tubular structures. There was no statistical difference of tubular and interstitial areas of the renal tissue between UUO-V and UUO-BBG groups. Sham animals did not show significant histological alterations (data shown in the supplementary material).

### P2X7R expression

**Fig. 1:**
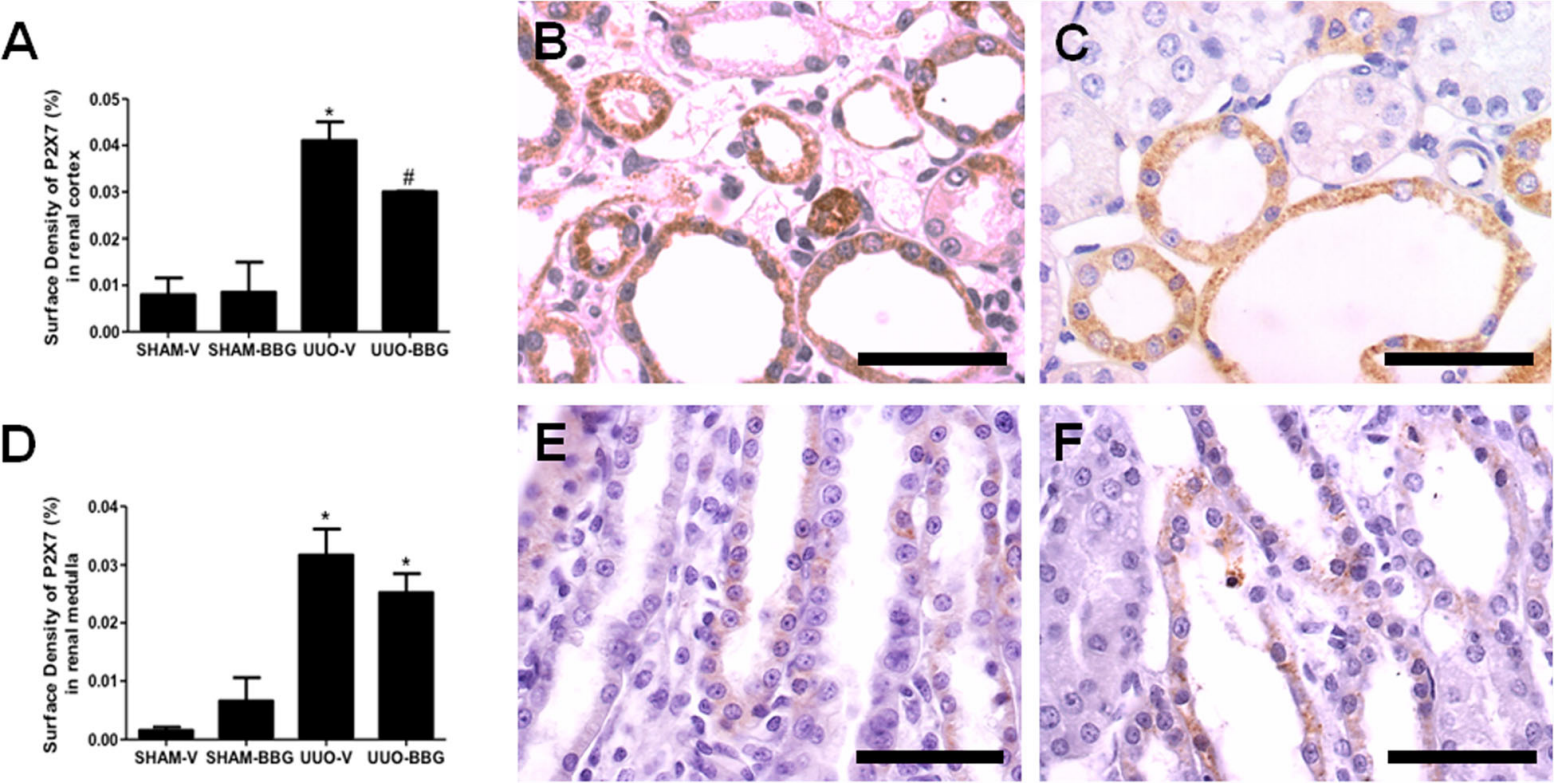
BBG administration decreases P2X7-R immunoexpression in tubular cortex but not in medulla in UUO. **a** Graphical representation of P2X7 surface density in tubular cells in renal cortex. The graphic shows aligned dot plot and the mean ± SEM of 20 captured images of each animal kidney in the different experimental groups. * *p* < 0.05 vs UUO-BBG and both SHAM groups. # *p* < 0.05 vs UUO-V and both SHAM groups. **b** Representative immunostaining of the renal cortex from UUO-V group. The arrows indicate one immunostained interstitial cell. **c** UUO-BBG group. **d** Graphical representation of P2X7 surface density in tubular cells in renal medulla. The graphic shows aligned dot plot and the mean ± SEM of 20 captured images of each animal kidney in the different experimental groups. * *p* < 0.05 vs both SHAM groups, but not between UUO groups. Representative immunostaining of the renal medulla from (**e**) UUO-V group and (**f**) UUO-BBG group. All pictures of the sham control groups are shown in the supplementary material. Bar = 50 μm in all figures.

Histomorphometrical analysis of P2X7R showed increased immunostaining in UUO groups compared to Sham groups in renal cortex (0.041 ± 0.004 vs 0.007 ± 0.004; UUO-V vs Sham-V, respectively; *p* < 0.05) and (0.03 ± 0.001 vs 0.008 ± 0.006, UUO-BBG vs Sham-BBG, respectively; *p* < 0.05). BBG decreased the surface density of the P2X7R in UUO in renal cortex (0.041 ± 0.01 vs 0.030 ± 0.001; UUO-V vs UUO-BBG, respectively, *p* < 0.05) (Fig. 1a), but not in renal medulla (0,031 ± 0,004 vs 0,025 ± 0,003; OUU-V vs OUU-BBG, respectively, *p* > 0.05) (Fig. 1d). UUO-V groups showed more immunoreactive tubules on renal cortex, but not on medulla, compared to UUO-BBG groups (Fig. 1). The sham control groups are shown in the Fig. 1 of the supplementary material (1A: Sham-V Cortex; 1B: Sham-BBG Cortex; 1C: Sham-V Medulla and 1D: Sham-BBG Medulla).

### BBG attenuates macrophage infiltration and downregulates the expression of IL-1β mRNA

**Fig. 2:**
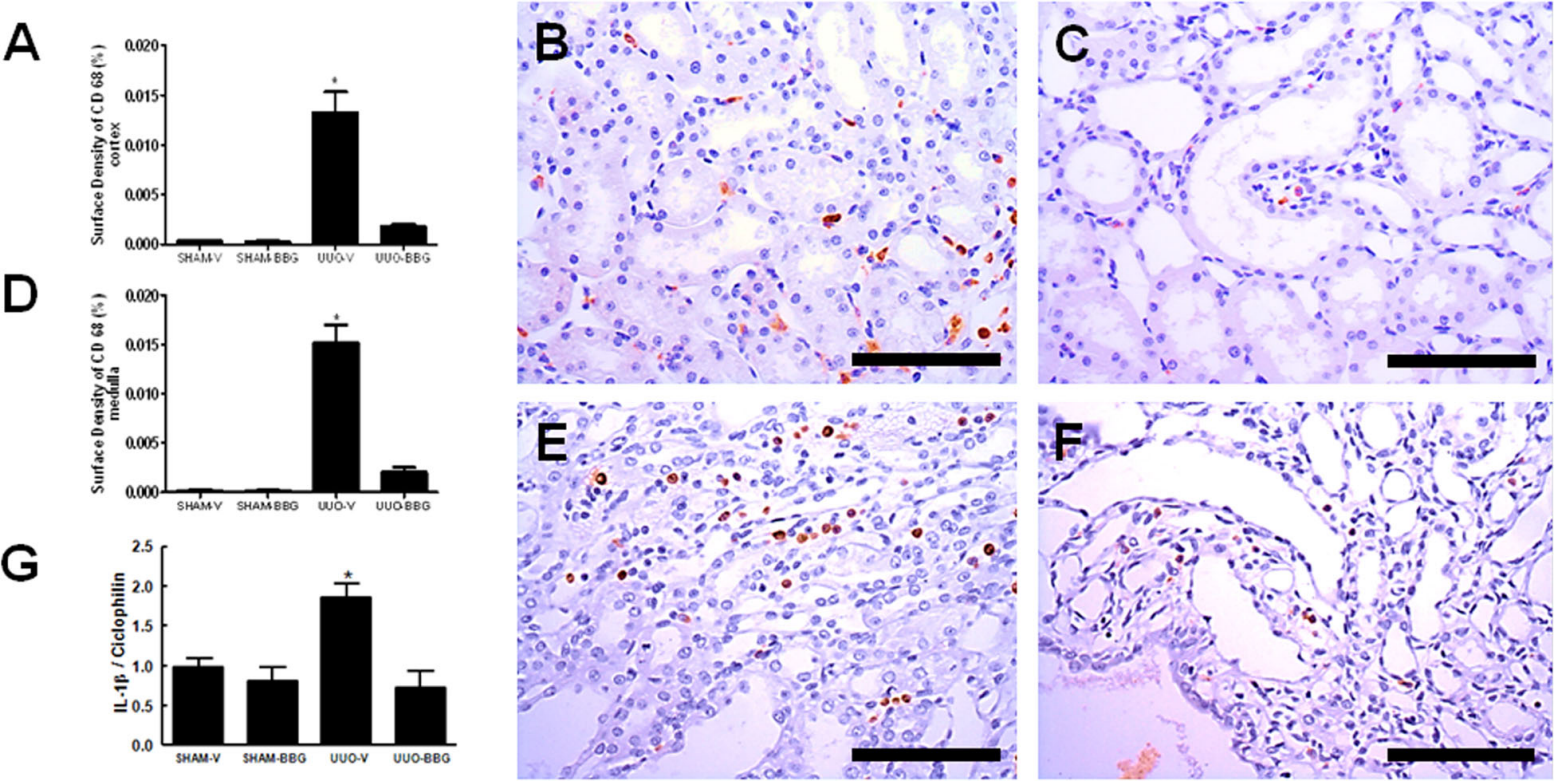
BBG and inflammation. BBG reduces the infiltration of macrophages in the renal interstitium and the mRNA expression of IL1-β in UUO. **a** Graphical representation of CD68 surface density in tubular cells in renal cortex. The graphic presents aligned dot plot and mean ± SEM of 20 captured images of each kidney in the different experimental groups. * *p* < 0.05 vs UUO-BBG and both SHAM groups. # *p* < 0.05 vs UUO-V and both SHAM groups. **b** Representative immunostaining of the renal cortex from UUO-V group. **c** UUO-BBG group. **d** Graphical representation of CD68 surface density in tubular cells in renal medulla. The graphic shows aligned dot plot and the mean ± SEM of 20 captured images of each kidney in the different experimental groups. * *p* < 0.05 vs UUO-BBG and both SHAM groups. # *p* < 0.05 vs UUO-V and both SHAM groups. Representative immunostaining of the renal cortex from (**e**) UUO-V group and (**f**) UUO-BBG group. All pictures of the sham control groups are shown in the supplement. Bar = 100 μm in all Figs. (**g**) Graphical representation of the mRNA expression of IL1-β by semiquantitative RT-PCR. Quantification of densitometric values obtained from the ratio IL1-β /Ciclophilin (mean ± SEM, *n* = 5) under the 3 experimental conditions indicated on the abscissa. * *p* < 0.05 vs UUO-BBG and both SHAM groups

Macrophages were immunostained with the anti-CD-68 antibody, and located in interstitial spaces, around glomerular capsule and blood vessels. UUO-BBG animals showed a decrease of about 85% of anti-CD-68 antibody surface density compared to UUO-V animals in renal cortex (0.013 ± 0.002 vs 0,003 ± 0; UUO-V vs UUO-BBG, respectively; *p* < 0.0001; Fig. 2a, b, c) and medulla (0.015 ± 0.003 vs 0.002 ± 0.001; UUO-V vs UUO-BBG, respectively; *p* < 0.0001; Fig. 2d, e, f). The sham control groups are shown in the Fig. 2 of the supplementary material (2A: Sham-V Cortex; 2B: Sham-BBG Cortex; 2C: Sham-V Medulla and 2D: Sham-BBG Medulla).

Tissue samples from UUO animals treated with BBG showed a reduction of about 60% of IL-1β mRNA expression compared to UUO-V animals (2.9 ± 0.8 vs 0.4 ± 0.08; UUO-V vs OUU-BBG, respectively; *P* < 0.0001; Fig. 2g).

### BBG attenuates myofibroblast infiltration and heat shock protein 47 expression

**Fig. 3:**
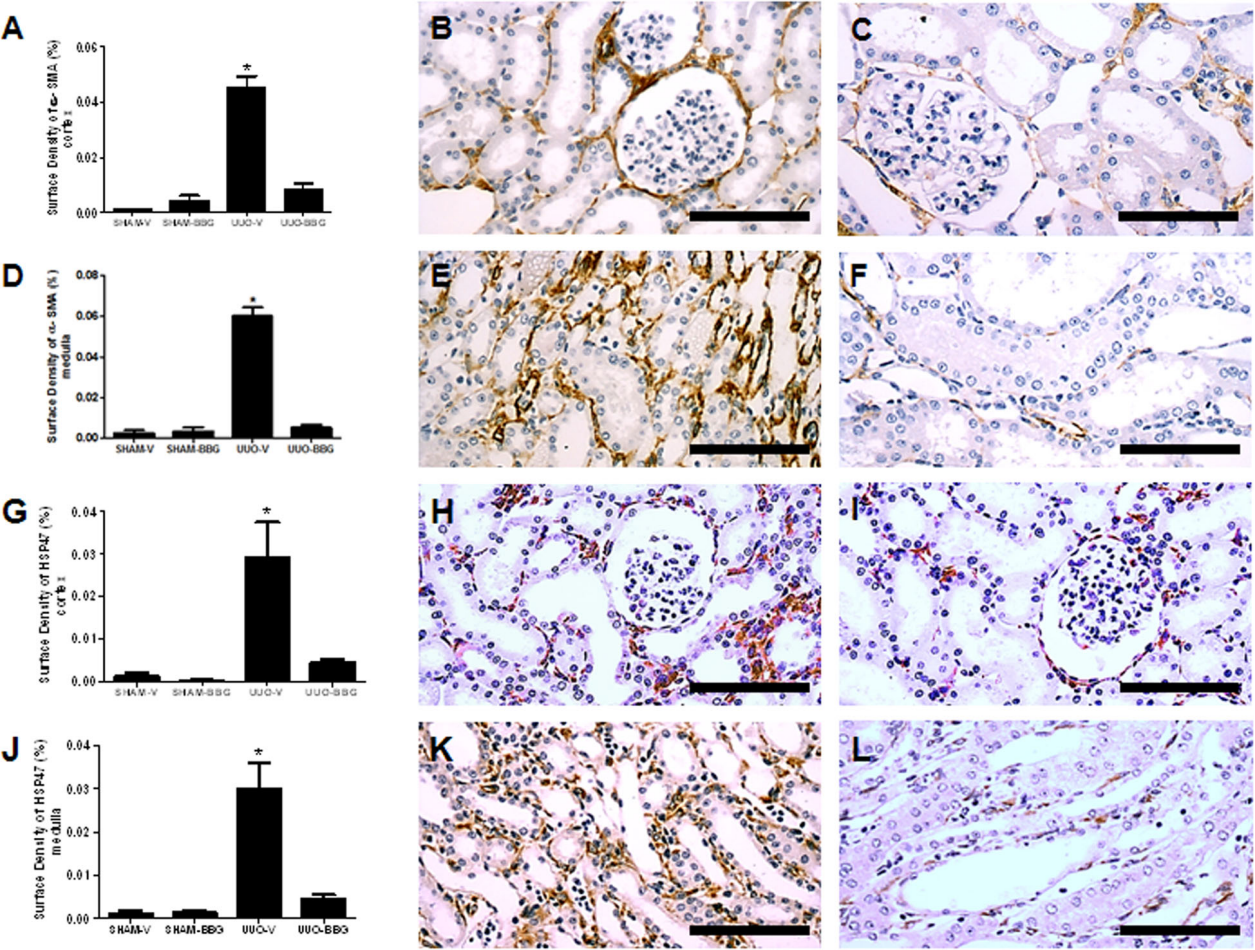
BBG reduces the pool of myofibroblasts and collagen synthesis in UUO. **a** Graphical representation of α-SMA surface density in renal cortex. The graphic presents aligned dot plot and the mean ± SEM of 20 captured images of each kidney in the different experimental groups. * *p* < 0.05 vs UUO-BBG and both SHAM groups. # *p* < 0.05 vs UUO-V and both SHAM groups. **b** Representative immunostaining of the renal cortex from UUO-V group and **c** UUO-BBG group. **d** Graphical representation of α-SMA surface density in renal medulla. The graphic shows aligned dot plot and the mean ± SEM of 20 captured images of each kidney in the different experimental groups. * *p* < 0.05 vs UUO-BBG and both SHAM groups. **e** Representative immunostaining of the renal medulla from UUO-V group and **f** UUO-BBG group. **g** Graphical representation of HSP-47 surface density in renal cortex. The graphic presents aligned dot plot and the mean ± SEM of 20 captured images of each kidney in the different experimental groups. * *p* < 0.05 vs UUO-BBG and both SHAM groups. **h** Representative immunostaining of the renal cortex from UUO-V group and **i** UUO-BBG group. **j** Graphical representation of HSP-47 surface density in renal medulla. The graphic presents aligned dot plot and the mean ± SEM of 20 captured images of each kidney in the different experimental groups. * *p* < 0.05 vs UUO-BBG and both SHAM groups. **k** Representative immunostaining of the renal medulla from UUO-V group and **l** UUO-BBG group. All pictures of the sham control groups are shown in the supplementary material. Bar = 100 μm in all figures

The surface density of α-smooth muscle actin in tissue samples from UUO-BBG animals showed about 80% reduction of myofibroblast surface density in renal cortex (0.045 ± 0.004 vs 0.007 ± 0.002; UUO-V vs UUO-BBG, respectively; *p* < 0.0001) and medulla (0.060 ± 0.3 vs 0.005 ± 0.001; UUO-V vs UUO-BBG, respectively; *p* < 0.0001), compared to UUO-V animals (Fig. 3a-f). The sham control groups are shown in the Fig. 3 of the supplementary material (3A: Sham-V Cortex; 3B: Sham-BBG Cortex; 3C: Sham-V Medulla and 3D: Sham-BBG Medulla).

HSP47 immunostaining was performed to analyze collagen synthesis. HSP47 immunostained area was reduced about 85% in UUO-BBG group compared to UUO-V animals in renal cortex (0.029 ± 0.008 vs 0.004 ± 0.001; UUO-V vs OUU-BBG, respectively; *P* < 0.0001) (Fig. 3g-i) and medulla (0.029 ± 0.7 vs 0.004 ± 0.001; UUO-V vs OUU-BBG, respectively; *P* < 0.0001) (Fig. 3j-l). The sham control groups are shown in the Fig. 4 of the supplementary material (4A: Sham-V Cortex; 4B: Sham BBG-Cortex; 4C: Sham-V Medulla and 4D: Sham-BBG Medulla).

### BBG attenuates collagen deposition and gene expression of procollagen I, III, and IV

**Fig. 4:**
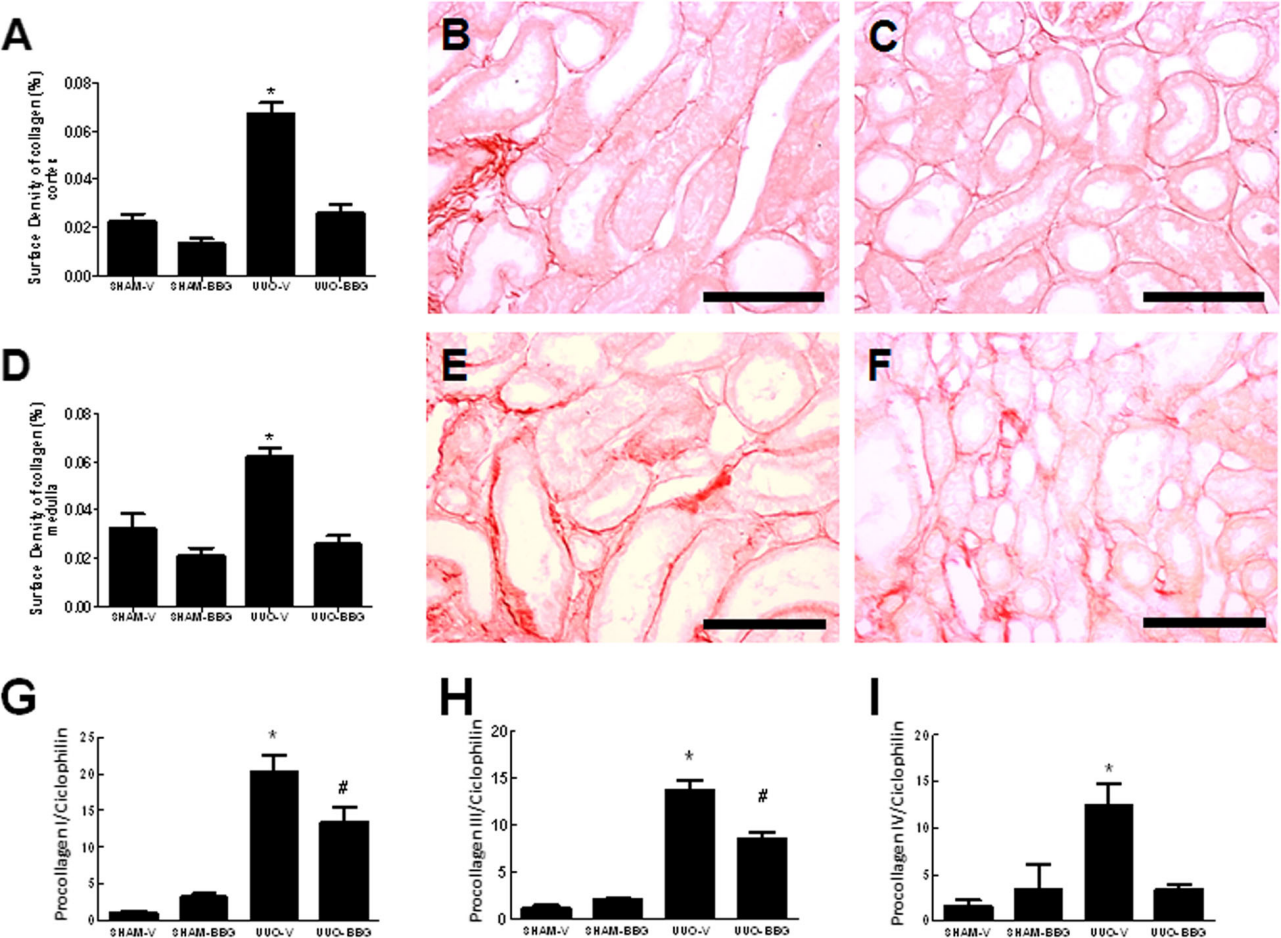
BBG reduces the renal collagen expression (protein and mRNA) in UUO. **a** Graphical representation of Picro-sirius Red surface density in renal cortex. The graphic shows aligned dot plot and the mean ± SEM of 20 captured images of each kidney in the different experimental groups. * *p* < 0.05 vs UUO-BBG and both SHAM groups. **b** Representative staining of the renal cortex from UUO-V group and **c** UUO-BBG group (Bar = 100 μm). **d** Graphical representation of Picro-sirius Red surface density in renal medulla. The graphic shows aligned dot plot and the mean ± SEM of 20 captured images of each kidney in the different experimental groups. * *p* < 0.05 vs UUO-BBG and both SHAM groups. **e** Representative staining of the renal medulla from UUO-V group and **f** UUO-BBG group. All pictures of the sham control groups are shown in the supplementary material. Bar = 100 μm in all Figs. (**g**) Graphical representation of the mRNA expression of Procollagen I by semiquantitative RT-PCR. Quantification of densitometric values obtained from the ratio Procollagen I /Ciclophilin (mean ± SEM, *n* = 6) under the 4 experimental conditions indicated in abscissa. * *p* < 0.05 vs UUO-BBG and both SHAM groups. # *p* < 0.05 vs UUO-V and both SHAM groups. **h** Graphical representation of the mRNA expression of Procollagen III by semiquantitative RT-PCR. Quantification of densitometric values obtained from the ratio Procollagen III/Ciclophilin (mean ± SEM, *n* = 5) under the 4 experimental conditions indicated in the abscissa. * *p* < 0.05 vs UUO-BBG and both SHAM groups. # *p* < 0.05 vs UUO-V and both SHAM groups. **g** Graphical representation of the mRNA expression of Procollagen IV by semiquantitative RT-PCR. Quantification of densitometric values obtained from the ratio Procollagen IV/Ciclophilin (mean ± SEM, *n* = 6) under the 4 experimental conditions indicated in the abscissa. * *p* < 0.05 vs UUO-BBG and both SHAM groups

The effect of P2X7R blockage on the early stage of UUO-induced collagen deposition in renal tissue was assessed by picro-sirius red staining. UUO-V animals showed the highest level of collagen compared to all other groups in renal cortex and medulla. UUO-BBG rats presented nearly 60% less collagen compared to UUO-V group, in renal cortex (0.067 ± 0.004 vs 0.025 ± 0.003; UUO-V vs UUO-BBG, respectively; *p* < 0.05) (Fig. 4a-c). The same pattern was observed in renal medulla (0.062 ± 0.004 vs 0.025 ± 0.003; UUO-V vs UUO-BBG, respectively; *p* < 0.05) (Fig. 4d-f). The sham control groups are shown in the Fig. 5 of the supplementary material (5A: Sham-V Cortex; 5B: Sham-BBG Cortex; 5C: Sham-V Medulla and 5D: Sham-BBG Medulla).

The qRT-PCR technique was performed to evaluate the expression of procollagen I, III and IV mRNA at day 3 of UUO. When comparing groups UUO-BBG and UUO-V, there was reduction of the expression of procollagen I mRNA (20.2 ± 2.4 vs 13.4 ± 2; UUO-V vs UUO-BBG, respectively; *p* = 0.0001) (Fig. 4g), and procollagen III mRNA (13.62 ± 1.1 vs 8.4 ± 0.75; UUO-V vs UUO-BBG, respectively; *p* < 0.0001) (Fig. 4h). Moreover, procollagen IV mRNA was reduced in about 70% in UUO-BBG compared to UUO-V group (12.4 ± 2.2 vs 3.4 ± 0.5; UUO-V vs UUO-BBG, respectively; *p* < 0.01) (Fig. 4i).

### BBG downregulates the immunoexpression of TGF-β

**Fig. 5:**
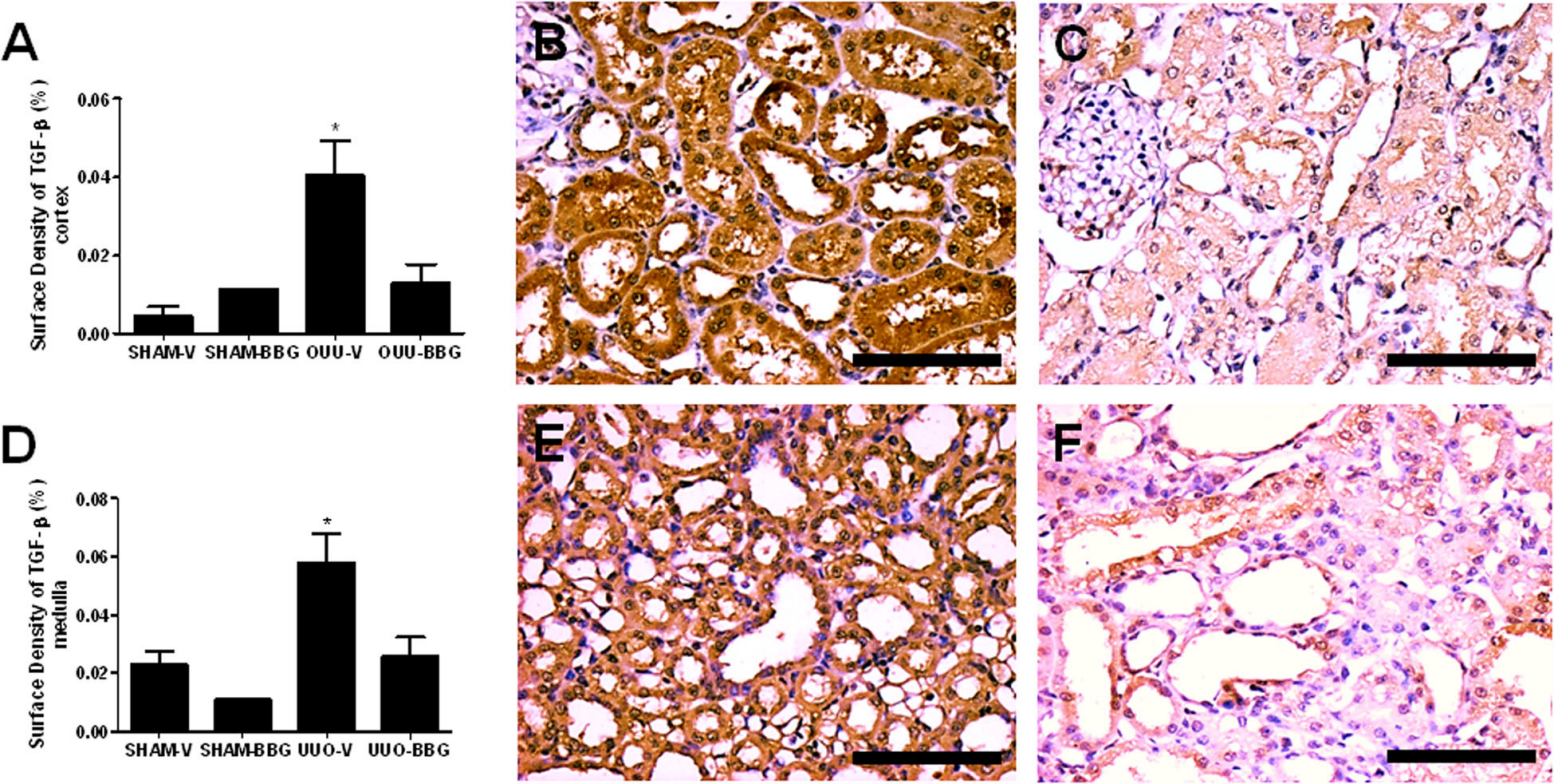
BBG reduces the immunoexpression of TGF-β in UUO. **a** Graphical representation of TGF-β surface density in renal cortex. The graphic presents aligned dot plot and mean ± SEM of 20 captured images of each kidney in the different experimental groups. * *p* < 0.05 vs UUO-BBG and both SHAM groups. # *p* < 0.05 vs UUO-V and both SHAM groups. **b** Representative immunostaining of the renal cortex from UUO-V group and **c** UUO-BBG group. **d** Graphical representation of TGF-β surface density in renal medulla. The graphic shows aligned dot plot and mean ± SEM of 20 captured images of each kidney in the different experimental groups. * *p* < 0.05 vs UUO-BBG and both SHAM groups. # *p* < 0.05 vs UUO-V and both SHAM groups. **e** Representative immunostaining of the renal medulla from UUO-V group and **f** UUO-BBG group. All pictures of the sham control groups are shown in the supplementary material. Bar = 100 μm for all figures

TGF-β was present in cortical and medullar renal tubules as well as in mononuclear inflammatory cells and vessels walls. Only the tubular TGF-β immunostaining was considered in the histomorphometric analysis. Renal tubules from UUO-BBG group showed a 75% reduction of TGF-β immunoexpression compared to UUO-V group in renal cortex (0.04 ± 0.01 vs 0.01 ± 0.010; OUU-V vs OUU-BBG, respectively; *p* = 0.0008) (Fig. 5a-c) and a reduction of about 60% in renal medulla (0.06 ± 0.01 vs 0.02 ± 0.01; OUU-V vs OUU-BBG, respectively; *p* < 0.05) (Fig. 5d-f). The sham control groups are shown in the Fig. 6 of the supplementary material (6A: Sham-V Cortex; 6B: Sham-BBG Cortex; 6C: Sham-V Medulla and 6D: Sham-BBG Medulla).

### BBG increases tubular cell regeneration and attenuates apoptosis

**Fig. 6:**
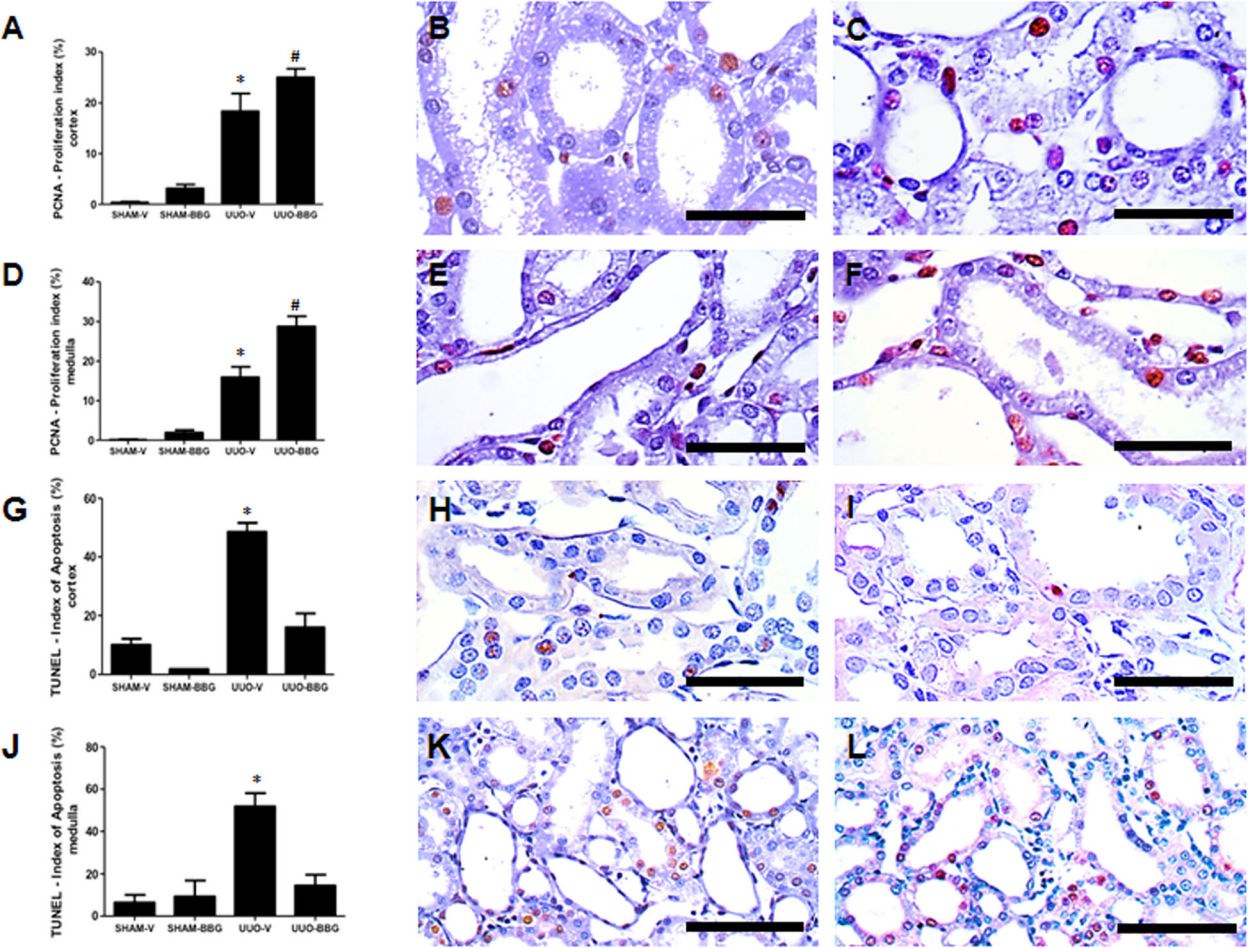
BBG administration induces proliferation and decreases apoptosis in tubular cells in UUO. **a** Graphical representation of a semi-quantitative analysis of the proliferation index (percentage of PCNA^+^ cells) in renal cortex and (**d**) medulla. The graphics show aligned dot plot and mean ± SEM of 20 captured images of each kidney in the different experimental groups. * *p* < 0.05 vs UUO-BBG and both SHAM groups. # *p* < 0.05 vs UUO-V and both SHAM groups. **b** Representative cortical immunostaining from UUO-V and **c** UUO-BBG (Bar = 50 μm). **e** Representative medullary immunostaining from UUO-V and (**f**) UUO-BBG (Bar = 50 μm). **g** Graphical representations of a semi-quantitative analysis of the apoptotic index (percentage of TUNEL^+^ cells) in renal cortex and (**j**) medulla. The graphics show aligned dot plot and mean ± SEM of 20 captured images of each kidney in the different experimental groups. * *p* < 0.05 vs UUO-BBG and both SHAM groups. **h** Representative cortical immunostaining from UUO-V and (**i**) UUO-BBG (Bar = 50 μm). **k** Representative medullary immunostaining from UUO-V and **l** UUO-BBG (Bar = 100 μm). All pictures of the sham control groups are shown in the supplementary material

The effect of BBG on proliferation and loss of tubular cells by apoptotic cell death at day 3 of UUO were evaluated by immunohistochemistry for the PCNA antigen and the TUNEL assay, respectively. UUO-BBG group showed significant higher tubular cell regeneration compared to UUO-V group in the cortex (about 35%) (18.4 ± 3.4 vs 25.0 ± 1.7; UUO-V vs UUO-BBG, respectively; *p* < 0.0001) and medulla (about 80%) (15.9 ± 2.68 vs 28.8 ± 2.6; UUO-V vs UUO-BBG, respectively; *p* < 0.05) (Fig. 6a-f). The sham control groups are shown in the Fig. 7 of the supplementary material (7A: Sham-V Cortex; 7B: Sham-BBG Cortex; 7C: Sham-V Medulla and 7D: Sham-BBG Medulla).

Furthermore, UUO-BBG group showed about 70% less tubular cell apoptosis compared to UUO-V group in the cortex (48.5 ± 3.08 vs 16 ± 4.78; UUO-V vs UUO-BBG, respectively; *p* < 0.0001), and medulla (51,8 ± 6,25 vs 14,5 ± 5,09 OUU-V vs OUU-BBG, respectively; *p* < 0.05), (Fig. 6g-l). The sham control groups are shown in the Fig. 8 of the supplementary material (8A: Sham-V Cortex; 8B: Sham-BBG Cortex; 8C: Sham-V Medulla and 8D: Sham-BBG Medulla).

## Discussion

Several lines of evidence suggest that purinergic P2X7 receptors participate in the processes of various kidney diseases [14–17]. In several previous studies, BBG, a selective P2X7R antagonist, was used as a potent inhibitor of P2X7R that reduces inflammation, immune cells activation, and fibrosis [10, 18, 19]. BBG was able to reduce renal injury in Dahl salt-sensitive hypertensive rats and in lupus nephritis model [6, 10] similarly to P2X7R null mice [5]. Of note, BBG also antagonizes rat P2X4Rs, but its selectivity for P2X7R is 1000-fold greater [20, 21]. The present study addressed the effects of BBG on the initial process of renal interstitial inflammation, collagen deposition, renal cell apoptosis and proliferation, by using the model of unilateral ureteral obstruction. Our results showed that BBG attenuated renal damage, similar to those in P2X7R knockout mice [5] and in other disease models [10, 14, 18, 19, 22, 23].

Our previous study about the effects of P2X7 receptors used the same fibrogenic model of renal disease [5]. In that study, we used knockout mice for P2X7R and we observed that these receptors could not be seen as constitutively expressed, but only in obstructed groups, probably localized on epithelial tubular cells. In addition, P2X7R were apparent only on day 7 of UUO and not on day 14. The present study was designed to observe the pathophysiological aspect of rat kidneys on day 3 of UUO, in which P2X7R was assumed to be expressed. In fact, P2X7R expression could be clearly seen by the immunohistochemical study, on the aspect of tubular epithelial cells, as well as in some interstitial cells. On the other hand, P2X7R were also expressed on sham-operated animals (Fig. 1 of the supplementary material), although with significantly lower intensity. It is noteworthy that their expression was seen in BBG treated UUO group as significantly lower when compared with non-treated UUO group, an observation also mentioned by Marques et al. [18] in another study using BBG. The explanation for the decreased expression of P2X7R on BBG treated group is not apparent at this moment, but one can argue about the possibility of receptor downregulation by BBG antagonism or allosteric modification of the epitope. Future studies are needed to elucidate this issue.

The results on the monocytes/macrophage recruitment after UUO clearly showed the expected increase of renal interstitial inflammation, which was decreased in BBG treated animals (Fig. 2). Nonetheless, perhaps the most specific feature associated with P2X7R activation and the development of tissue inflammation might be the secretion of inflammasome-related cytokines [24, 25]. In this regard, it has been consistently documented that the effect of P2X7R activation is closely linked to IL-1β secretion [24, 26]. In renal tissue, Deplano et al. [16] and Jalilian et al. [27] have previously demonstrated the role of ATP, as a damage-associated molecular pattern, on the activation of P2X7R to trigger the secretion of IL-1β. Our results showed that the increased IL-1β mRNA in UUO tissue kidneys was abrogated in BBG-treated animals (Fig. 2g).

A significant reduction in myofibroblast population (Fig. 3a-f) was observed in BBG treated group. Moreover, an effect on myofibroblast function was strongly suggested from the results of HSP47 (Fig. 3g-l). This chaperone is an endoplasmic reticulum (ER)-resident, stress inducible glycoprotein, collagen-specific heat-shock protein, which plays a key role in collagen biosynthesis and its structural assembly [28]. It is also used as a biomarker to identify collagen-producing cells [29]. A previous study using the model of UUO in mice clearly showed that HSP-47 was overexpressed in the renal interstitium of obstructed animals [30]. In our study, BBG treated group showed decreased expression of the chaperone. Therefore, it is conceivable to suggest that the reduction of HSP-47 stained cells probably indicates a reduction of myofibroblasts function by the purinergic blockage.

The aspect of interstitial collagen deposition is a known striking histopathological feature of the UUO model. In this study, the expression of TGF-β, the fibrogenic cytokine associated with collagen deposition, was shown to be decreased in BBG treated rats (Fig. 5). Also, decreased collagen deposition, as expressed by picrosirius red staining, was seen in BBG treated group (Fig. 4a-f). Likewise, pro-collagen I, III and IV mRNA were shown to be increased in obstructed animals, with significant reduction in BBG treated rats (Fig. 4g-i). It is also noteworthy that experiments done in our laboratory using 14 days UUO in rats, also revealed the effect of BBG on collagen deposition in both cortex and medulla (Figs. 9 and 10 of the supplementary material).

Our previous study on P2X7 knockout mice clearly evidenced the implication of these receptors on the process of apoptosis of renal cells [5]. In fact, the present study showed the decrease of apoptotic cells in kidneys from BBG treated group. Likewise, renal cells proliferation also increased in the group with BBG, and these results suggest that regenerative process after kidney damage by UUO can be up-regulated by P2X7R antagonism (Fig. 6). We have previously demonstrated the action of bone marrow-derived cells to induce and attenuate renal cell proliferation and apoptosis, respectively [31]. Nevertheless, little is known about the involvement of P2X7 receptors activation in this setting. It is noteworthy, however, that Chen et al. [32] have found an increase in P2X7R expression after derangement of retinal ganglion cells, which decreased in animals treated with human umbilical cord blood mesenchimal stem cells. This observation suggests a mechanistic antagonism between P2X7R expression and activation, and the action of progenitor cells to determine proliferative repair. In the present study, the putative BBG action to inhibit P2X7R activation, which was related to increased proliferation of renal cells, might suggest a modulatory role of this receptor on the mechanism of renal cell repair after epithelial cell injury.

## Conclusion

The results from this study highlight the beneficial role of P2X7R antagonism, as can be accomplished by BBG, in the prevention of the early phase of inflammation and the ensuing fibrogenic process, even in the third day of UUO. As the previous studies using P2X7R antagonists in various disease models, one can suggest that targeting this receptor might be beneficial in selective conditions. In addition, this study also constitutes evidence that the blockage of purinergic P2X7 receptor may act in favor of renal cell proliferation and tissue regeneration, and the mechanism underlying this effect needs extensive investigation.

## Supplementary information

**Additional file 1.**

**Additional file 2.**

**Additional file 3.**

**Additional file 4.**

**Additional file 5.**

**Additional file 6.**

**Additional file 7.**

**Additional file 8.**

**Additional file 9.**

**Additional file 10.**

## Data Availability

The datasets analysed during the current study are available from the corresponding author on reasonable request (mleitejr@gmail.com).

## Abbreviations

BBG: Brilliant blue G
UUO: Unilateral ureteral obstruction
PCNA: Proliferating Cell Nuclear Antigen
TUNEL: Terminal deoxynucleotidyl transferase dUTP nick end labeling
qRT-PCR: Reverse transcription polymerase chain reaction quantitative real time
IL-1β: Interleukin 1 beta
mRNA: Messenger ribonucleic acid
ATP: Adenosine triphosphate
CKD: Chronic kidney disease
DMSO: Dimethyl sulfoxide
