# Supplementary figures and images for: Brilliant blue G, a P2X7 receptor antagonist, attenuates early phase of renal inflammation, interstitial fibrosis and is associated with renal cell proliferation in ureteral obstruction in rats
Source: BMC Nephrol. 2020 May 29;21:206. doi: 10.1186/s12882-020-01861-2 (PMC7260756; doi:10.1186/s12882-020-01861-2)

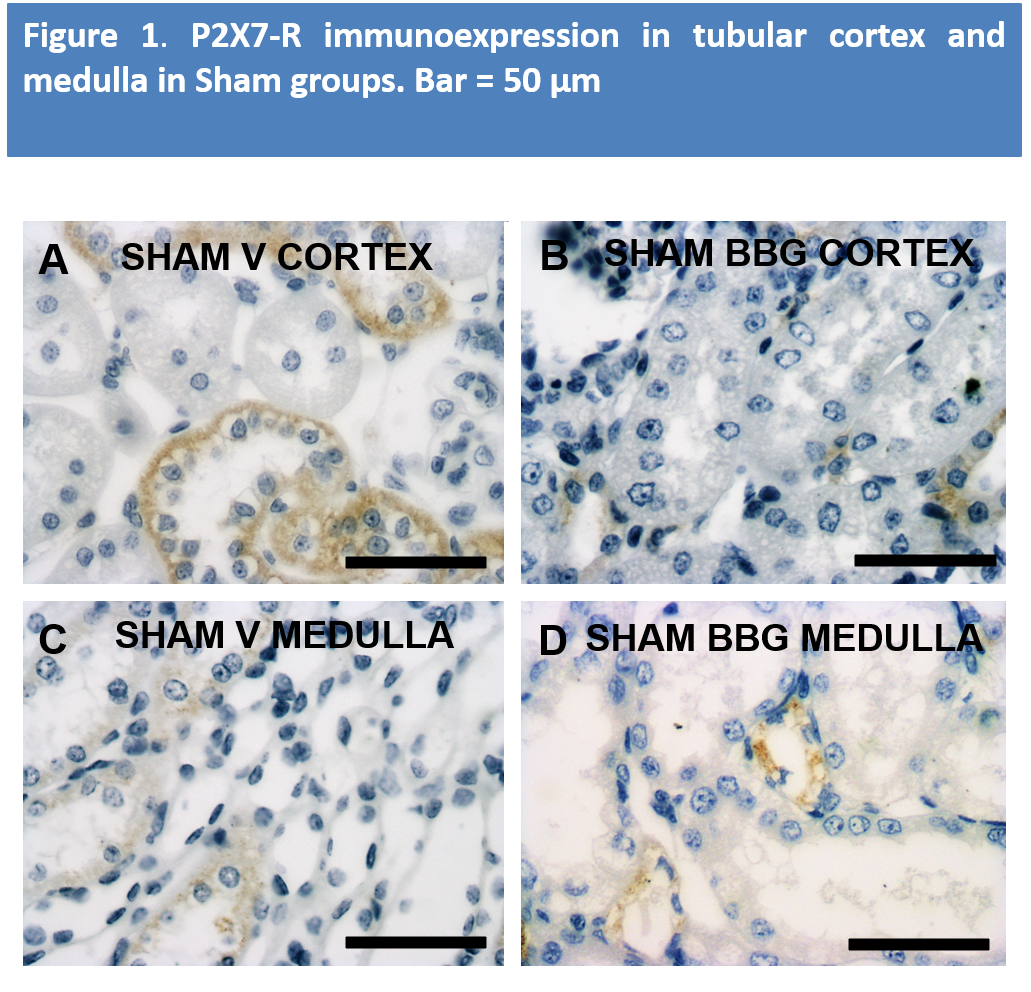

Supplement: Supplementary file 1 — Additional file 1. [file 12882_2020_1861_MOESM1_ESM.tif]

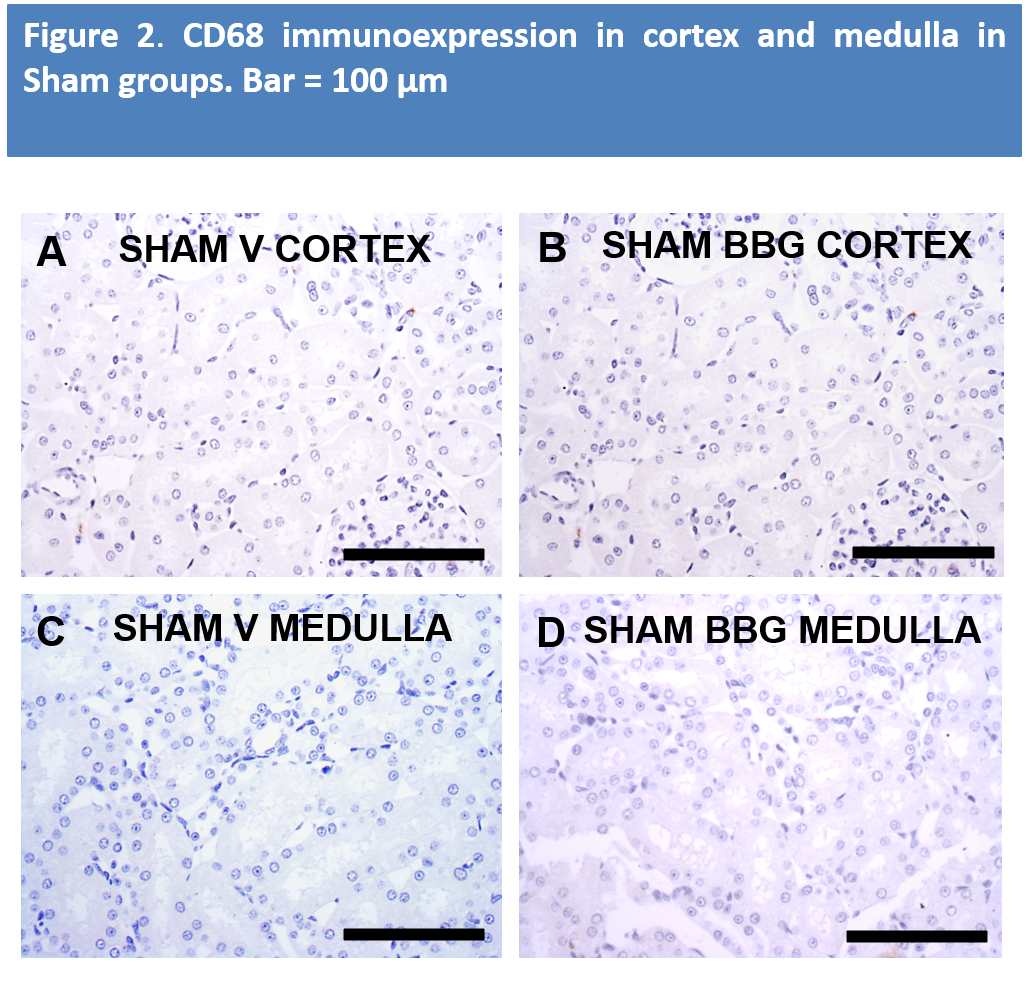

Supplement: Supplementary file 2 — Additional file 2. [file 12882_2020_1861_MOESM2_ESM.tif]

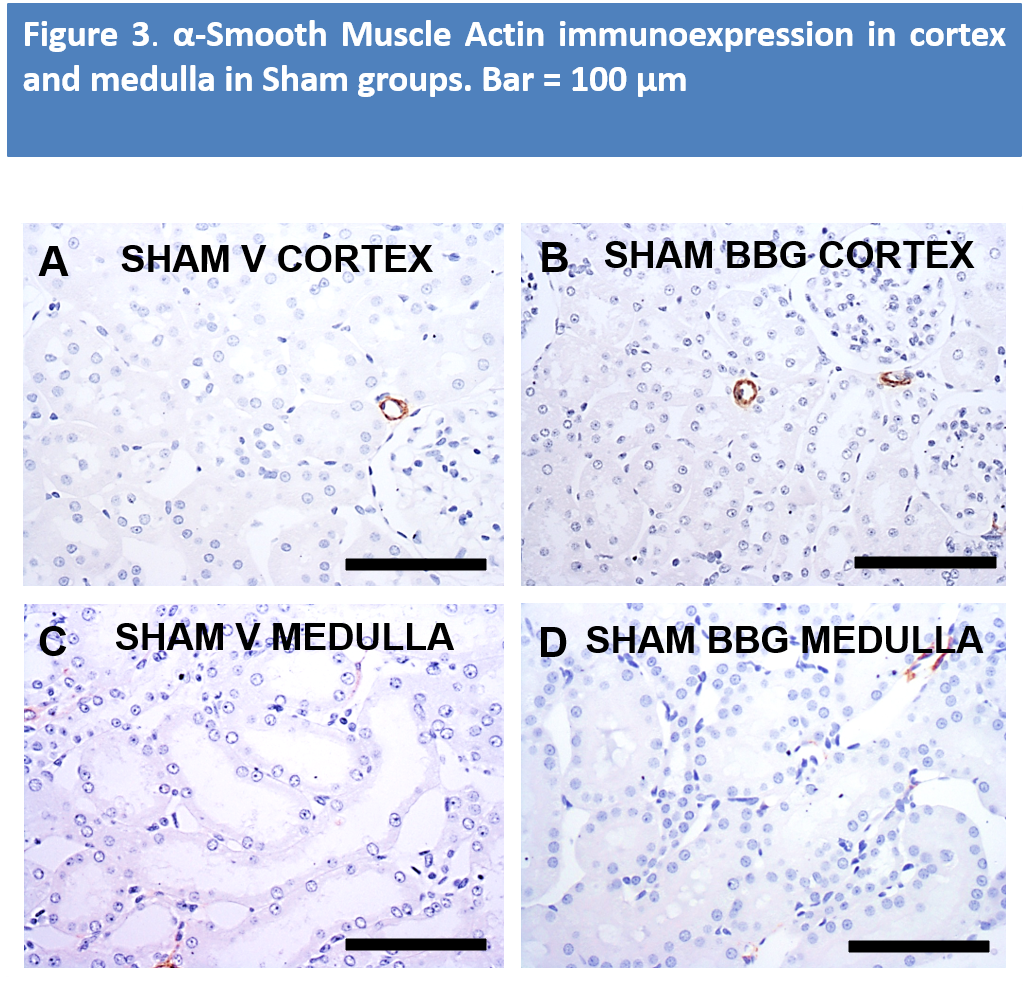

Supplement: Supplementary file 3 — Additional file 3. [file 12882_2020_1861_MOESM3_ESM.tif]

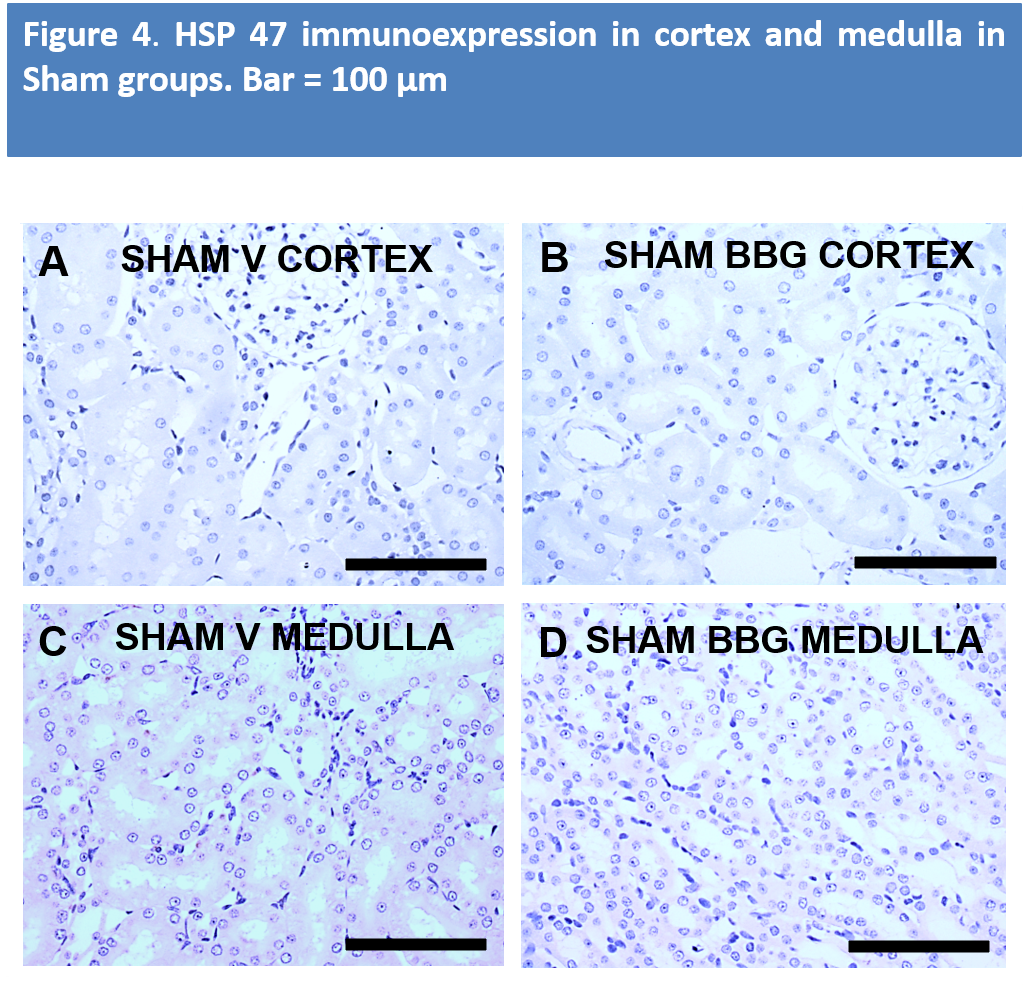

Supplement: Supplementary file 4 — Additional file 4. [file 12882_2020_1861_MOESM4_ESM.tif]

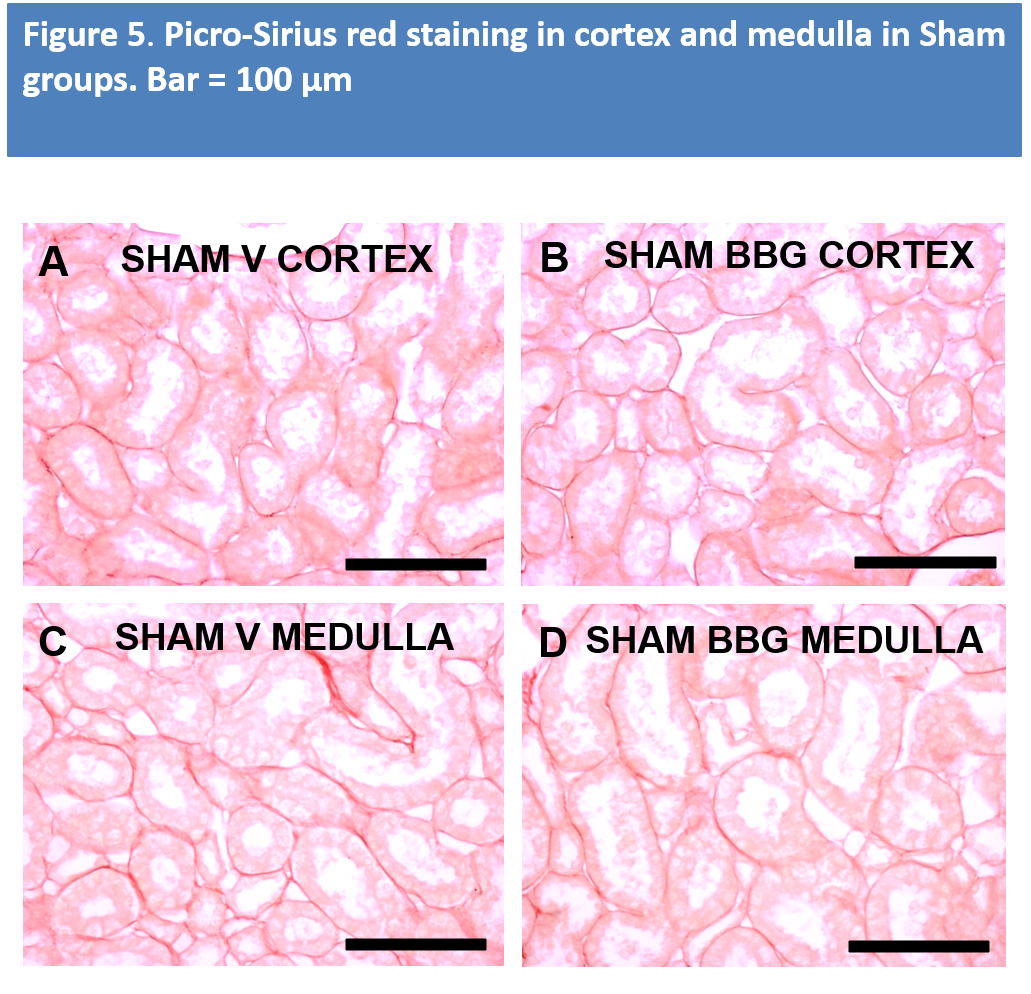

Supplement: Supplementary file 5 — Additional file 5. [file 12882_2020_1861_MOESM5_ESM.tif]

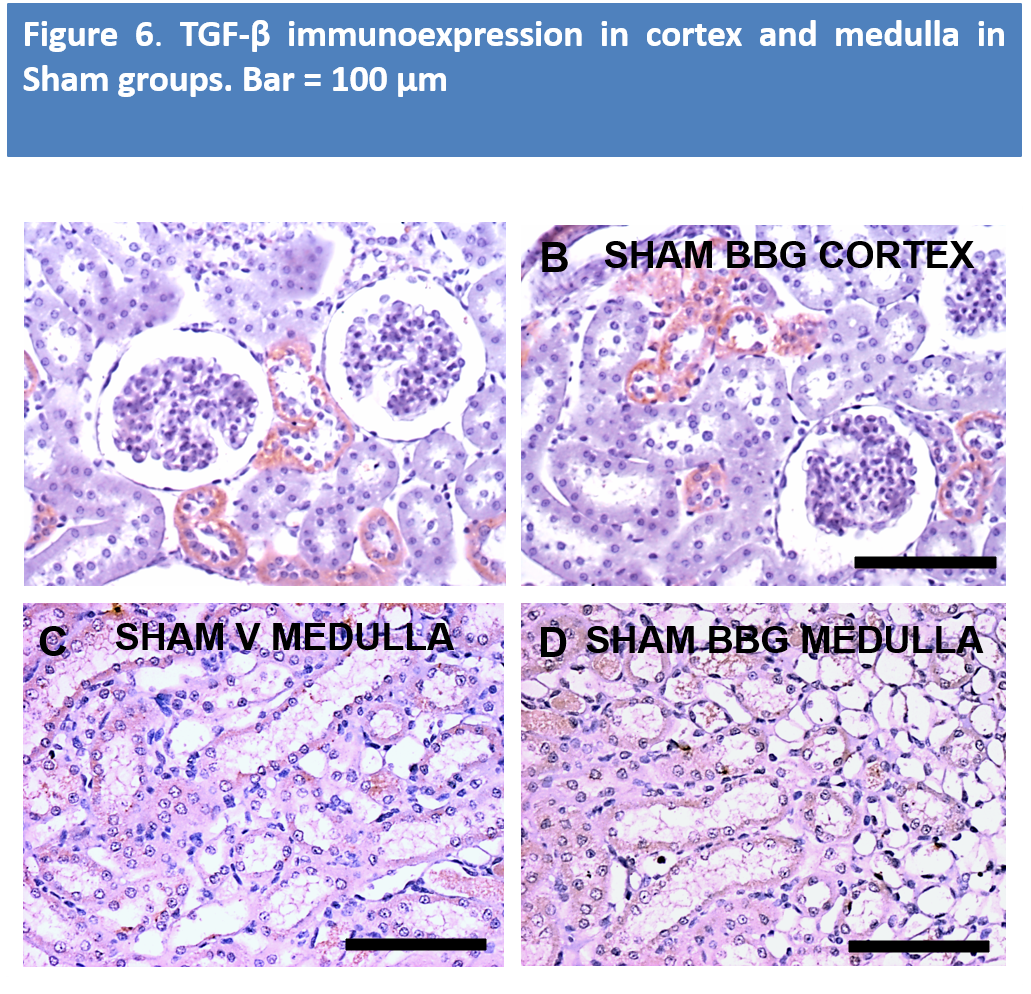

Supplement: Supplementary file 6 — Additional file 6. [file 12882_2020_1861_MOESM6_ESM.tif]

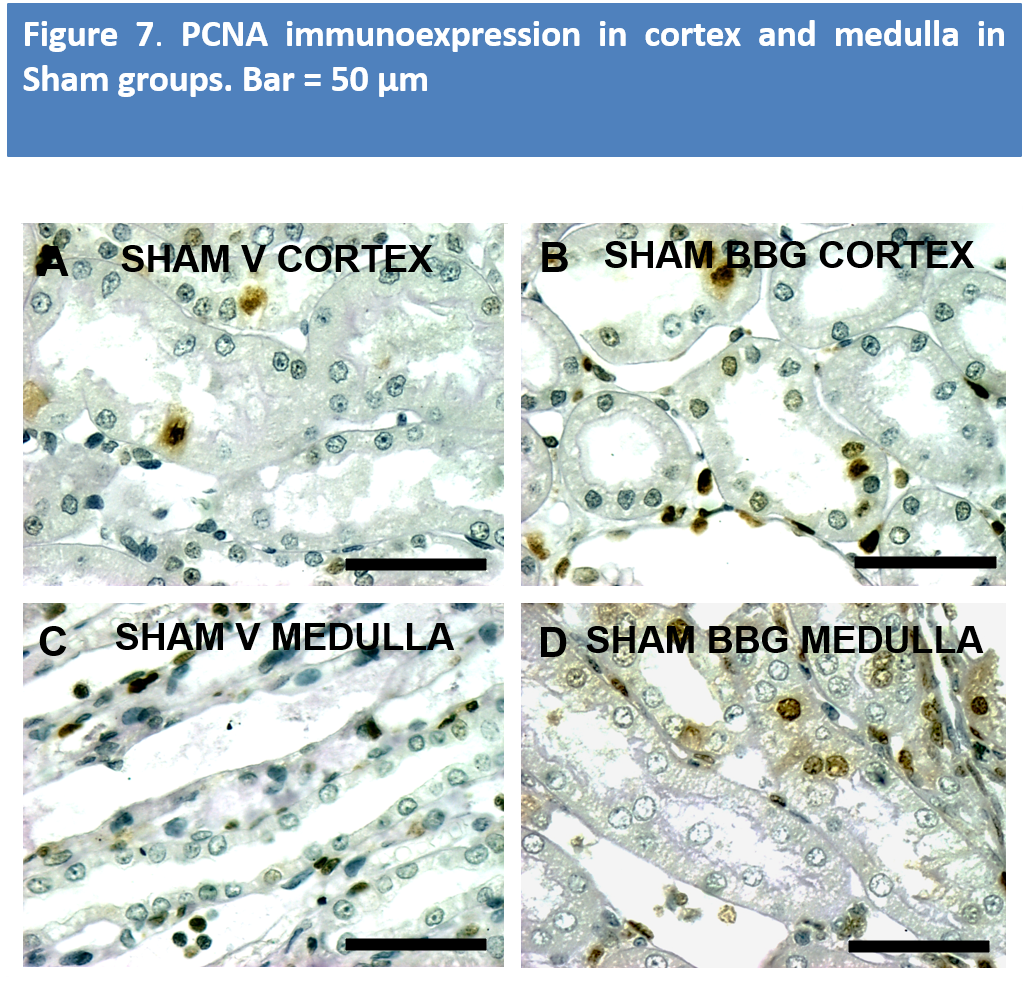

Supplement: Supplementary file 7 — Additional file 7. [file 12882_2020_1861_MOESM7_ESM.tif]

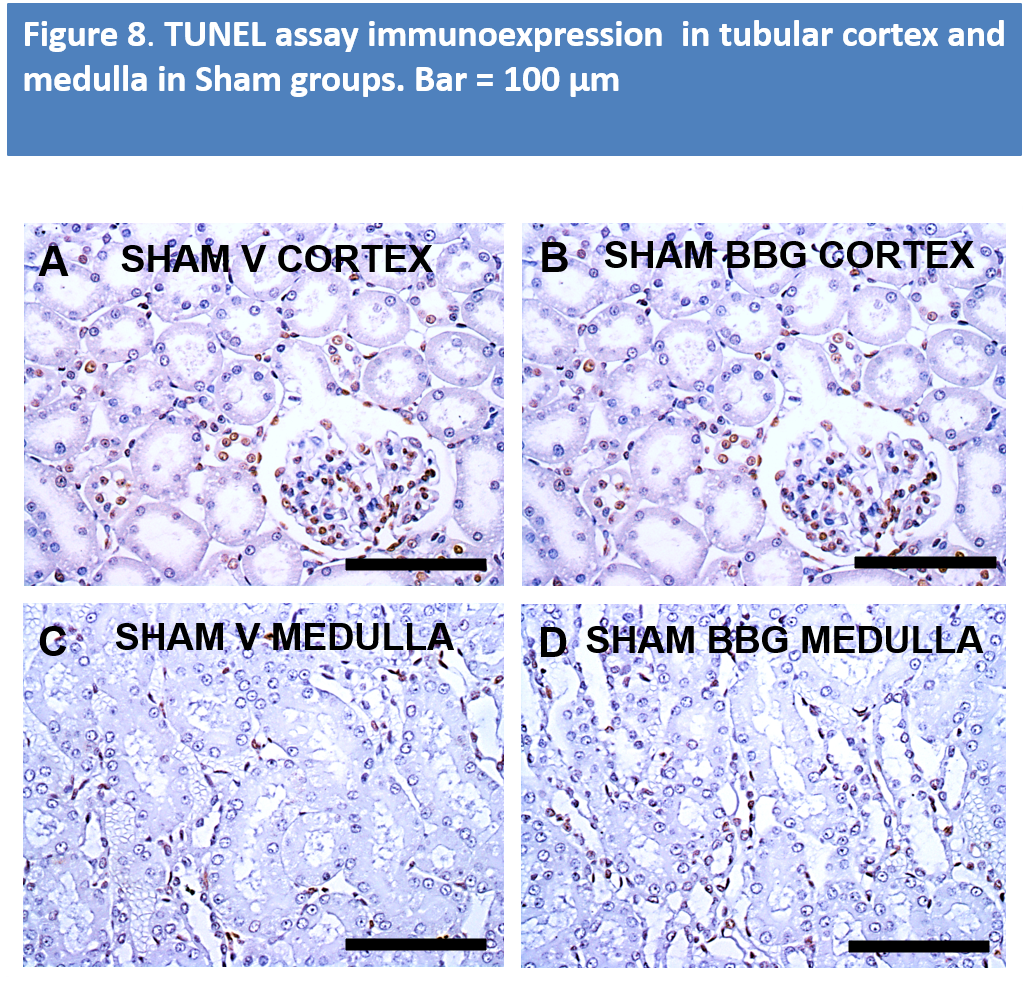

Supplement: Supplementary file 8 — Additional file 8. [file 12882_2020_1861_MOESM8_ESM.tif]

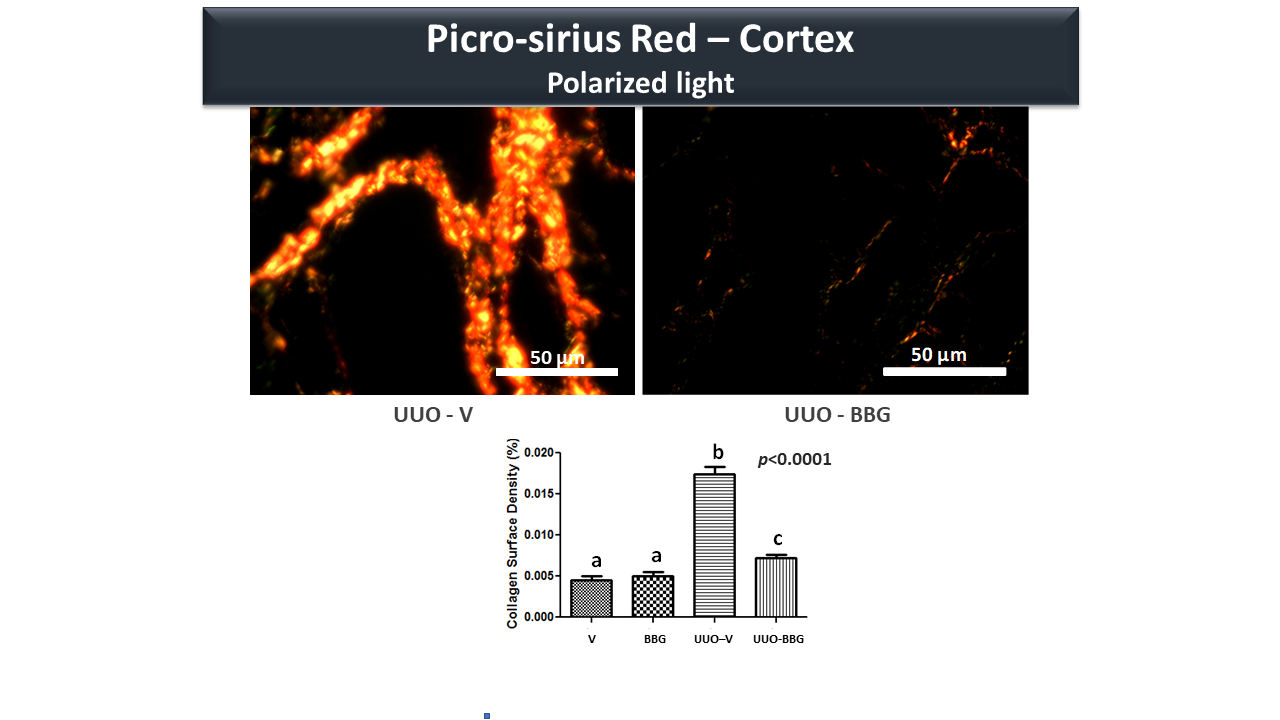

Supplement: Supplementary file 9 — Additional file 9. [file 12882_2020_1861_MOESM9_ESM.tif]

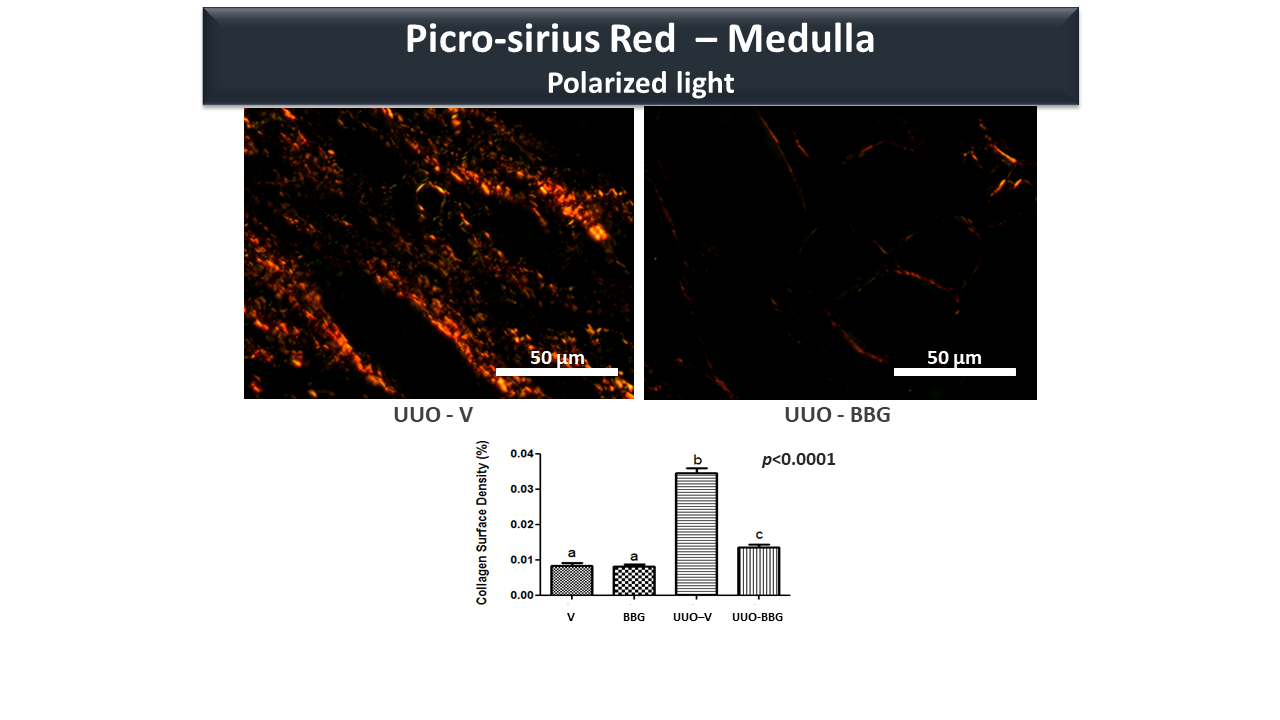

Supplement: Supplementary file 10 — Additional file 10. [file 12882_2020_1861_MOESM10_ESM.tif]
